# Mortality in Elderly Patients Taking Furosemide: Prospective Cohorts Study

**DOI:** 10.1155/2022/4708259

**Published:** 2022-10-29

**Authors:** Alejandro Rodríguez-Molinero, Antonio Miñarro, Leire Narvaiza, César Gálvez-Barrón, Natalia Gonzalo León, Esther Valldosera, Eva De Mingo, Oscar Macho, David Aivar, Efren Pinzón, Adilis Alba, Jorge Passarelli, Nadia Stasi, Isabel Collado, José R. Banegas

**Affiliations:** ^1^Consorci Sanitari De L'Alt Penedès I Garraf, Sant Pere De Ribes, Barcelona, Spain; ^2^Department of Genetics, Microbiology and Statistics, Faculty of Biology, University of Barcelona, Barcelona, Spain; ^3^ACE Alzheimer Center Barcelona (Fundació ACE), Barcelona, Spain; ^4^Fundació Privada Sant Antoni Abat, Vilanova i La Geltrú, Barcelona, Spain; ^5^Department of Preventive Medicine and Public Health, Universidad Autónoma de Madrid – CIBER in Epidemiology and Public Health (CIBERESP), Madrid, Spain

## Abstract

**Objectives:**

Low blood pressure (BP) has been proposed as a risk factor of death in elderly patients. However, this association could be partially accounted for by the deleterious effects of BP-lowering drugs. We analyzed whether these drugs are associated to an increased risk of death in elderly patients taking multiple potential confounders into account.

**Design:**

This is a prospective cohort study. *Setting and Participants.* Probabilistic sample of 772 community-dwelling patients aged >65 years living in Spain, who were appointed for an initial clinical visit and followed up through telephone calls 4, 6, 9, 12, and 60 months afterwards.

**Methods:**

At baseline visit, BP was measured using standardized methods, and BP medications and risk factors of death in elderly patients (BMI, oxygen saturation, toxic habits, comorbidity, muscular strength, and functional and cognitive capacity) were collected. During the follow-up, the vital status of patients and the date of death were ascertained.

**Results:**

During a median 5-year follow-up, 226 all-cause deaths occurred among the 686 participants included in the analysis. In a Cox regression model that included all the BP drug classes, diuretics and nitrites were significantly associated with mortality (*p* < 0.005). Within diuretics, furosemide was found to be responsible for the association of the group. In multivariable Cox regression models adjusted for BP and the rest of the mortality risk factors, furosemide remained as the only BP drug that was independently associated with mortality (hazard ratio 2.34; *p* < 0.01).

**Conclusions:**

Furosemide was prospectively associated with increased mortality in older people. If confirmed, this drug should be taken into account by prescribers and considered a confounder in BP studies.

## 1. Introduction

High systolic and diastolic blood pressure (BP) has been associated with increased mortality in the general population [1, 2], although studies in elderly patients have yielded conflicting results. A number of observational studies have shown a “*J*-shaped” or “*U*-shaped” distribution of the risk of death associated with BP values [3–8]. This finding means that the risk in the older population increases both with high and excessively low BP values, and in some of these studies, “reverse causality” (i.e., BP decreases close to death) was reasonably excluded. However, the few clinical trials conducted on elderly people up to date have failed to show such a mortality increase with low BP values [9–11]. The HYVET study demonstrated an important mortality reduction in the intervention group, where the target BP had been established in 150/80 mmHg or lower values [11]. The SPRINT study demonstrated that a target systolic BP < 120 mmHg was associated with a lower rate of fatal and nonfatal cardiovascular events, as compared with a target systolic BP < 140 mmHg [9].

A major difference between clinical trials and observational studies is the different ability to control for several confounders. Few observational studies have made careful adjustments of BP-lowering drugs as possible confounders in the BP-mortality relationship; most of them included drug consumption dichotomously in adjusted models (i.e., presence or absence of antihypertensive drugs) [3, 5, 6] or, in some cases, presence or absence of certain drug-classes like diuretics [12]. Such analyses seem to be insufficient to exclude possible deleterious effects of BP-lowering drugs on survival, which could potentially act as confounding factors in observational studies.

The low BP-related mortality increase in elderly subjects might be due, at least partially, to the harmful effects of some of the BP-lowering drugs they take. In this study, we analyze whether BP-lowering drugs are associated with an increased risk of death in elderly patients taking several potential confounders of this relationship into account.

## 2. Methods

This study is based on data from the cohort of elderly people of the Consorci Sanitari del Garraf [13]. We included 772 community-dwelling elderly people (older-than-65-years individuals) [14], who were appointed for a baseline clinical visit and subsequently followed up through telephone calls 4, 6, 9, 12, and 60 months afterwards. We conducted a multistage probabilistic sampling of the Spanish territory (towns, districts within towns, and homes within districts) using door-to-door contact and telephone calls. The sample was stratified by sex, age, town size (rural community, urban community, big city), and geographical zone (Northeast, Southeast, Southwest, and Northwest of Spain) and included a nonproportional age stratum with overrepresentation of subjects older than 79 years because, according to the researchers' criterion, they are highly representative of the particular physiological characteristics of the elderly.

In order to reduce nonsampling errors, the interview was carefully designed, data collectors were adequately trained (through theoretical and practical sessions), and the field work, surveys, data coding, and data processing were carefully supervised. To minimize lack of response, a candidate was replaced for another one only in case of 10 failed contact attempts, failure to attend 2 scheduled visits, refusal or inability to participate, institutionalization, or death. In the field work, data collectors were accompanied by a supervisor during their first survey visits. Additionally, a telephonic control of 15% of the included patients was made, to assess aspects related to the inclusion of the patient in the study, verify the veracity of their responses, and check the procedure used to take physical measurements such as blood pressure. All the completed questionnaires were reviewed by a team, who was also in charge of retrieving all missing data through telephone calls, whenever possible. This quality control detected 100 patients in the cohort, who had not been selected through the established probabilistic procedures and were consequently excluded.

During the baseline visit, systolic and diastolic BP was measured with a validated electronic device (Omron HEM-7000-E), after 5-minute rest in the sitting position. BP was measured twice, and the first value was excluded from the analysis. Besides BP, the following variables were recorded: age, sex, the complete list of used BP medications (active ingredient and daily dose), toxic habits (smoking and alcohol consumption), and disease background, including hypertension and cardiac diseases (ischemic heart disease and other cardiac diseases), according to the questions of the Spanish National Health Survey [15]. Comorbidity was calculated on the basis of the recorded chronic diseases: cardiovascular (hypertension, stroke, ischemic heart disease, and other cardiac diseases), diabetes mellitus, hyperlipidemia, respiratory diseases (asthma, chronic bronchitis), allergy, Parkinson's disease, migraine and other neurological diseases (grouped), peptic ulcer, neoplasia, anemia, thyroid diseases, prostate diseases, osteoporosis, chronic skin problems, depression, urinary incontinence, constipation, and degenerative joint diseases (osteoarthritis, low back pain, hip and/or knee prosthesis). Cognitive capacity was also evaluated using the Pfeiffer scale [16]. Functional capacity was evaluated through the Katz questionnaire, which assesses the patient's independence for bathing, dressing, toileting, transferring, feeding, and continence (scores range from 0 to 6, where 6 indicates dependency for all activities) [17]. Muscle strength of the upper and lower limbs was measured through the Medical Research Council (MRC) scale, which ranges from 0 to 5 for every muscle group, where 5 corresponds to normal strength [18]. Finally, patients' height and weight were measured in order to calculate their body mass index (BMI), and peripheral oxygen saturation was measured with a finger pulse oximeter (9500; Nonin Medical, Plymouth, MN).

Mortality (all-cause deaths) was assessed through follow-up telephone calls, in which the date of death was asked to a relative or another close person. The mortality data of 202 patients that had been lost to follow-up were recovered from the Spanish National Institute for Statistics, which provided information on their vital status after 5 years.

Informed consent was obtained from all the participants before being included in the study. The research protocol was previously approved by the local Ethical Committee.

### 2.1. Statistical Analysis

The statistical analysis was conducted in 2 phases. In the first phase, the association between the risk of death and different BP-lowering drug classes (ACE inhibitors, ARBs, beta-blockers, alpha-blockers, calcium antagonists, nitrovasodilators, and diuretics) was studied both in a univariate manner and in a joint multivariate analysis, in which all pharmacological groups were introduced as independent variables (logistic regression model in which variables were selected by the Lasso method) [19]. For the analysis, drug use was dichotomized: use vs. no use at baseline.

In the second phase, multivariate analysis models were conducted (Cox regression) with mortality as a dependent variable, where relevant drugs were introduced according to the results of the first phase (using *p* < 0.005), together with relevant BP-related variables, according to a previous univariate analysis (diastolic BP, pulse pressure) and risk factors of death recorded during the baseline visit (age, sex, smoking habit, cardiac diseases, comorbidities, functional capacity, cognitive capacity, and muscular strength). The risk factors introduced as covariables in the multivariate models were selected through the Lasso method, with leave-one-out cross-validation (LOOCV) [19]. Drugs remaining significant in the final models were further studied through survival analysis: Kaplan–Meier curves with log-rank test, adjusted for the rest of risk factors [20].

Finally, we analyzed the distribution of risk factors in cases lost to follow-up versus cases with complete follow-up, as well as in patients lost to follow-up who were taking drugs found to be relevant in the above analysis versus those who were not taking them.

We used software *R* version 3.5.0 [21], glmnet package [22] for the statistical analysis.

## 3. Results

**Figure 1:**
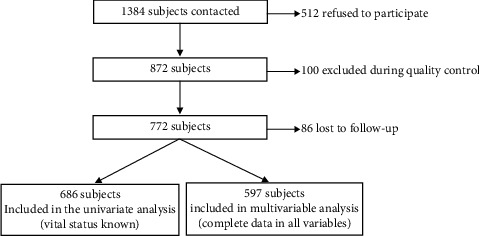
Flow chart of the sample.

The response rate of individuals invited to participate was 63%. Of the 686 patients included in the analysis, 226 participants died during the follow-up period, which corresponded to 33.0% (median follow-up, 5 years).  Figure 1 shows the data corresponding to recruitment and losses to follow-up.  Table 1 shows the characteristics of participants finally included in the analysis (mean age, 81.4 years, 63.3% women, mean BMI, 28.7 kg/m^2^) and the causes of death of the deceased participants. Total number of deaths and causes of death are summarized in Table 2.

ACE inhibitors, nitrites, and diuretics were associated with higher mortality in the univariate analysis (*n* = 686; Table 3). In a multivariate logistic model, where all drug classes were introduced as independent variables, the following ones showed independent significant association with mortality: diuretics (odds ratio [OR] 1.96; 95% confidence interval [CI]: 1.36–2.83) and nitrites (OR 3.09; 95% CI: 1.44–6.63). Given that “diuretics” include a variety of heterogeneous molecules, a second multivariate logistic model was made (*n* = 506), introducing the different diuretic drugs as independent variables. In this second analysis, nitrites remained in the final model (OR 2.56 95% CI: 1.02–5.43), as well as furosemide (OR 4.48; 95% CI: 2.47–8.12), which suggests that they were the main variables accounting for the association between diuretics and mortality.

**Figure 2:**
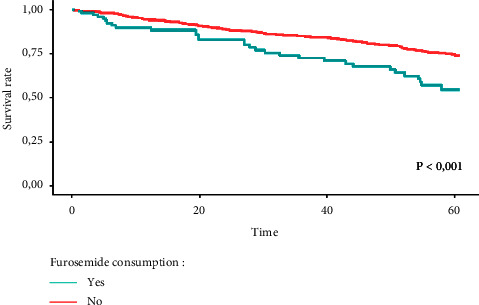
Survival in elderly persons exposed and not exposed to furosemide (adjusted for other risk factors of death: age, sex, smoking, functional capacity, muscle strength, comorbidity, cognition, heart disease, diastolic blood pressure, pulse pressure).

Nitrites and furosemide were then introduced into corresponding multivariate models using Cox regression, which were adjusted for other risk factors of death. Factors selected as covariates were as follows: age, sex, smoking habit, functional capacity (Katz index), muscular strength, comorbidity, cognitive capacity (Pfeiffer index), cardiac disease, diastolic BP, and pulse PP. As shown in Table 4, furosemide remained in the final multivariate model as a significant risk factor of mortality (hazard ratio [HR] 2.31, *p* < 0.01). Nitrites did not contribute to the risk independently of the other studied factors.  Figure 2 shows the survival curve corresponding to furosemide-exposed and not-exposed patients, adjusted for the other risk factors that were included as covariables in the Cox regression. The difference in survival between both groups was significant, according to the log-rank test (*p* < 0.0001).

To explore the possibility of a spurious association between furosemide and mortality due to imbalanced loss of patients at risk of death in both groups, we analyzed the differences in baseline characteristics and distribution of the different risk factors of death between patients lost to follow-up and patients who completed the study, as well as between patients lost to follow-up who were taking furosemide versus those who were not taking it. Patients lost to follow-up were older (*p* < 0.01) had more comorbidities (*p* < 0.001), less strength (*p* < 0.01), lower cognitive capacity (*p* < 0.05), and less independence for activities of daily living (Katz) (*p*=0.01) than those that completed the study. They also had lower mean diastolic BP (*p* < 0.05). Among patients lost to follow-up, those who were taking furosemide had more cardiac diseases (*p* < 0.05), higher BMI (*p*=0.01), more comorbidities (*p* < 0.05), than those who were not taking furosemide. These data evidence a higher risk of death among patients lost to follow-up who were treated with furosemide. Thus, it is unlikely that the main results are due to a selective loss of furosemide-exposed survivors.

## 4. Discussion

This study reveals an increased risk of death at 5 years among elderly patients who have been taking furosemide; this association is independent from the rest of the studied factors, including blood pressure and cardiac diseases.

Furosemide and other diuretics are extensively used in elderly patients (25–40% of patients older than 65 years) since they are usually prescribed to treat conditions that are highly prevalent in this population: hypertension, heart failure, and renal failure [23, 24]. Thus, a furosemide-associated increase in the risk of death could have important consequences, even for a relatively small risk increase, which does not seem to be the case.

Furosemide-associated increased risk of death has been described in patients with congestive heart failure, where the risk of death seems to grow with the dose and is independent of the severity of the disease [25–27]. Increased mortality was also described in elderly people with dementia, taking furosemide together with risperidone, although no specific pattern was identified for this association in terms of the cause of death [28]. We failed to find studies analyzing the association between furosemide and the risk of death in the general elderly population.

A number of studies have demonstrated a lack of association between high BP and the risk of death in the elderly population. Some studies even reported higher mortality among older adults with low BP values. However, it should be evaluated whether such an increased mortality with lower BP could actually be due to the negative effects of hypotensive drugs like furosemide. This hypothesis is especially relevant in the case of furosemide since the mentioned mortality increase has only been noted in observational studies, though not in clinical trials, where furosemide is not often used because it is not a first-line treatment for hypertension. In the HYVET clinical trial (N 3845; mean age, 83.6 years) [11], where higher mortality was not demonstrated for hypertensive elders with strict systolic and diastolic BP control, patients received indapamide and perindopril, instead of furosemide. In the SPRINT study (N 2636; mean age, 79.9 years), where no *J*-curve was found for the association between the risk of death and systolic BP, the prescription was free but thiazide diuretics were prioritized, as indicated in clinical guides [9]. In a *post-hoc* analysis of the SHEP clinical trial (N 4736; mean age, 72 years), conducted on hypertensive older adults treated with chlorthalidone and atenolol, the mortality increase associated with low BP was not statistically significant [10].

Conversely, most observational studies, conducted on the general elderly population, show a *J*-shaped association curve between mortality and BP values. Such studies involve a variety of hypotensive treatments, a factor that is usually roughly controlled or not controlled at all [3, 5, 6]. Noticeably, a rough control of this factor made the *J*-shape disappear in the Leiden 85-plus study [29] (N 271; mean age, 90 years) and reduced it in the Health & Retirement Study (N 7492; mean age, 74, 4 years) [5]. In the PARTAGE study (N 1126; mean age, 88 years), conducted on hypertensive institutionalized older adults, it was demonstrated that both hypotension and polypharmacy accounted for higher mortality in patients with systolic BP < 130 mmHg (after an important adjustment) [30]; noticeably, 67% of patients in the group of higher mortality (lower BP and use of multiple drugs) used loop diuretics, while in the other groups (monotherapy and not-low BP), the proportion of patients using these drugs ranged between 19% and 46% (this factor was not corrected). The SENIORS (N 2128; mean age, 76, 1 year) study, conducted on elderly patients with heart failure, demonstrated higher mortality for lower systolic BP [8]; noticeably, 85% of patients in this study were taking diuretics, and since these patients suffered from heart failure, a large part of them were most probably being treated with furosemide. The ZODIAC study (N 881; mean age, 73, 1 year), conducted on diabetic patients older than 60 years, showed a *J*-shaped association curve between the risk of death and systolic and diastolic blood BP values, although the shape disappeared in the group of nonhypertensive patients (who were probably receiving less hypotensive drugs) [31].

The results of our study lead us to postulate that furosemide might act as a confounder factor in studies of hypotension-associated mortality in older adults, given that its possible effects on mortality have not been controlled in statistical analyses. However, in our analysis, the risk of death associated with furosemide seems to be independent from that associated with diastolic hypotension. In this view, furosemide intake might not account completely for the *J*-shaped curve observed in other studies for the risk of death. The results of the Leiden 85-plus study are in this line since higher mortality with lower systolic BP is still observed in a group of patients who were not taking antihypertensive drugs [29].

Our study has several limitations that lead us to interpret our results cautiously. First, because of the small sample size, the potential risk associated with certain drugs or drug classes might have remained undetected. The subgroup of furosemide users dying during the study was also small in size, which did not preclude the statistical significance of results, and therefore, our results are probably not random, although it would be wise to verify them in larger samples. Second, our control for cardiac diseases was based on patients' self-reported data; thus, it may be inaccurate, and moreover, we have no information about the degree of severity of the reported diseases. Also, renal failure may act as a confounder factor, which we could not control for. However, this is unlikely to have happened, since the causes of death could be recorded, and only one user of furosemide died of kidney-related diseases. In addition, in some studies on furosemide-associated mortality in patients with congestive heart failure, where renal failure was actually controlled, the results did not change [26, 27]. Furthermore, furosemide might also cause or worsen renal failure; thus, the net effect of this risk factor is not easy to establish [32]. Finally, it is worth mentioning that hypokalemia that occurs with low-dose diuretics has been associated with a reduced benefit on cardiovascular events [33]. Therefore, hypokalemia could act as a confounding factor. However, since furosemide is a potassium-wasting diuretic, hypokalemia could be also an element in the causal chain, facilitating death in furosemide consumers. In the latter case, adjusting the models for this variable would not be advisable, as it could pose an overadjustment.

The amount of cases lost to follow-up in our study was not negligible and might cause spurious associations in case, for any reason, furosemide users completing the study suffered higher mortality than furosemide users lost to follow-up. To investigate such possibility, we analyzed the available risk factors of death affecting patients lost to follow-up. In general, such patients had more risk factors of death than patients who completed the study, and among patients lost to follow-up, furosemide users had more risk factors than control patients. This finding makes a selective loss of furosemide-user survivors improbable, as well as the occurrence of such a bias. In any event, the patients lost to follow-up, as a whole, had a higher risk of death than the patients retained in the study; therefore, patients at higher risk were underrepresented in the data analyzed, and the results may not be applicable to this group.

In conclusion, furosemide seems to increase mortality in elderly people, which if confirmed, would have important health implications for the elderly population. Furthermore, if this effect is not controlled, it could bias the results of observational studies on mortality associated with BP in the elderly.

## Figures and Tables

**Table 1:** Basal characteristics and causes of death of the sample included in the analysis.

	Total (686)	Alive (*n* = 460)	Deceased (*n* = 226)	*p*	No furosemide (*n* = 630)	Furosemide (*n* = 56)	*p*
Women	434,00	302 (65.7%)	132 (58.41%)	0.773	397 (63.0%)	37 (66.1%)	0.773

No tobacco	488,00	335 (73.1%)	153 (67.7%)	0.445	449 (71.5%)	39 (69.6%)	0.445

No alcohol	455,00	293 (63.7%)	162 (72.3%)	0.303	414 (65.9%)	41 (73.2%)	0.303

High BP	353,00	236 (51.3%)	117 (51.77%)	0.405	321 (51.0%)	32 (57.1%)	0.405

No heart disease	503,00	**350 (76.1**%**)**	**153 (67.7**%**)**	**<0.001**	**482 (76.5**%**)**	**21 (37.5**%**)**	**<0.001**

Age	81.39 (7.1)	**79.5 (6.9)**	**85.3 (5.7)**	**<0.001**	**81.2 (7.1)**	**83.9 (6.5)**	**0.002**

Body mass index	28.7 (4.8) 28.2 (IQR = 5.7)	28.8 (4.8) 28.3 (IQR = 5.5)	28.4 (4.9) 27.9 (IQR = 6.5)	0.350	28.6 (4.7) 28.2 (IQR = 5.6)	29.5 (6.2) 27.9 (IQR = 6.5)	0.641

Katz index	1.1 (1.8) 0 (IQR = 1)	**0,6 (1.3) 0 (IQR** **=** **1)**	**2.0 (2.3) 1 (IQR** **=** **4)**	**<0.001**	**1.0 (1.8) 0 (IQR** **=** **1)**	**1.9 (2.3) 0.5 (IQR** **=** **4)**	**0.007**

Muscle strength	28.8 (8.4) 33 (IQR = 7)	**30.4 (6.6) 34 (IQR** **=** **4)**	**25.2 (10.6) 30 (IQR** **=** **15)**	**<0.001**	**29.1 (8.1) 33 (IQR** **=** **6)**	**24.6 (10.7) 29 (IQR** **=** **18)**	**0.004**

Comorbidity	3.4 (2.1) 3 (IQR = 3)	3.3 (2.0) 3 (IQR = 3)	3.4 (2.3) 3 (IQR = 3)	0.697	**3.3 (2.0) 3 (IQR** **=** **3)**	**4.4 (2.5) 4 (IQR** **=** **5)**	**<0.001**

Pfeiffer index	1.7 (2.1) 1 (IQR = 2)	**1.2 (1.6) 1 (IQR** **=** **2)**	**2.6 (2.8) 1.5 (IQR** **=** **4)**	**<0.001**	**1.6 (2.1) 1 (IQR** **=** **2)**	**2.6 (2.8) 1 (IQR** **=** **4)**	**0.013**

Systolic BP	141.3 (22.8) 140 (IQR = 28)	141.9 (22.2) 140 (IQR = 28.8)	140.2 (24.0) 140 (IQR = 27.3)	0.365	**141.8 (22.6) 140 (IQR** **=** **27)**	**136.1 (25.0) 134 (IQR** **=** **27.0)**	**0.007**

Diastolic BP	79.8 (13.4) 80 (IQR = 18)	**81.0 (12.77) 81 (IQR** **=** **17)**	**77.3 (14.2) 77 (IQR** **=** **19)**	**<0.001**	**80.2 (13.4) 80 (IQR** **=** **17)**	**75.6 (12.7) 75.5 (IQR** **=** **19.3)**	**0.015**

PP	61.5 (18.9) 59 (IQR = 24.8)	60.8 (18.3) 59 (IQR = 23)	62.8 (20.1) 59.0 (IQR = 29.3)	0.346	61.6 (18.7) 59 (IQR = 25)	60.4 (21.2) 56.5 (IQR = 23.8)	0.401

O2 saturation	94.8 (3.8) 96(IQR = 3)	**95.2 (3.3) 96 (IQR** **=** **3)**	**94.0 (4.5) 95 (IQR** **=** **4)**	**0.002**	94.9 (3.76) 96 (IQR = 3)	93.9 (3.9) 95 (IQR = 5)	0.076

Data presented are *n* (%) or mean (SD) or median (interquartile range–IQR). ^*∗*^BP: blood pressure; PP: pulse pressure; comorbidity: number of diseases. Numbers in bold mean statistical significance.

**Table 2:** Total number of deaths and causes of death.

	Total (686)	No furosemide (*n* = 630)	Furosemide (*n* = 56)
Total	226 (32.9%)	**188 (29.8%)**	**38 (67.9%)**
Stroke	28 (4.1%)	25 (15.2%)	3 (8.3%)
Cardiovascular	41 (6.0%)	31 (19.3%)	10 (27.7%)
Respiratory	25 (3.6%)	19 (11.8%)	6 (16.6%)
Digestive system	5 (0.7%)	3 (1.9%)	2 (5.6%)
Renal	4 (0.6%)	3 (1.9%)	1 (2.8%)
Neoplasm	42 (6.1%)	36 (22.4%)	6 (16.7%)
Dementia	11 (1.6%)	9 (5.6%)	2 (5.6%)
Other	41 (6.0%)	35 (22.7%)	6 (16.7%)

Numbers in bold mean statistical significance (<0.001).

**Table 3:** Relationship between drug exposure and mortality at 5 years (univariate analysis).

	Deceased exposed	Deceased not exposed	Odds ratio	95% CI	*p*
ACE inhibitors	56 (41%)	170 (31%)	1.52	1.02–2.26	**0.042**
ARB	39 (30%)	187 (34%)	0.87	0.56–1.33	0.533
Beta-blockers	22 (30%)	204 (33%)	0.87	0.50–1.48	0.693
Calcium antagonists	32 (30%)	194 (33%)	0.88	0.55–1.34	0.652
Alpha-blockers	17 (44%)	209 (32%)	1.62	0.84–3.15	0.199
Nitrites	20 (61%)	206 (32%)	3.33	1.63–7.14	**0.001**
Diuretics	86 (45%)	140 (28%)	2.04	1.43–2.93	**0.001**
Torasemide	21 (41%)	205 (32%)	1.47	0.79–2.69	0.216
Spironolacton	13 (54%)	213 (32%)	2.49	1.09–6.00	**0.044**
Chlorthalidone	1 (11%)	225 (33%)	0.25	0.01–1.63	0.283
Hydrochlorothiazide	18 (38%)	208 (33%)	1.24	0.65–2.31	0.525
Indapamide	4 (31%)	222 (33%)	0.90	0.26–2.89	1.000
Amiloride	6 (46%)	220 (33%)	1.76	0.58–5.79	0.373
Furosemide	38 (68%)	188 (30%)	4.95	2.77–9.09	**<0.001**

**Table 4:** Multivariate models of mortality at 5 years.

*N* = 597	HR	IC 95%	*p*
Age	**1.10**	**1.07–1.13**	**<0.01**
Sex (men vs. women)	**1.96**	**1.32–2.91**	**<0.01**
Smoking (yes vs. no)	0.94	0.70–1.26	0.66
Functional capacity (Katz index)	**1.14**	**1.02–1.26**	**0.02**
Muscle strength	0.99	0.97–1.01	0.17
Comorbidity	1.07	0.98–1.16	0.14
Cognition Pfeiffer index	**1.13**	**1.05–1.22**	**<0.01**
Heart disease	0.94	0.69–1.28	0.69
Diastolic blood pressure	**0.99**	**0.98–1.00**	**0.03**
Pulse pressure	1.00	0.99–1.01	0.91
Furosemide (yes vs. no)	**2.31**	**1.48–3.61**	**<0.01**
Nitrites (yes vs. no)	0.40	0.86–2.57	0.16
Furosemide—nitrites (interaction)	0.87	0.29–2.58	0.80

## Data Availability

Data are available from the Consorci Sanitari de l'Alt Penedès-Garraf (contact: recerca@csapg.cat) for researchers who meet the criteria for access to confidential data.
